# Effect of spineboard and headblocks on the image quality of head CT scans

**DOI:** 10.1007/s10140-016-1396-z

**Published:** 2016-04-18

**Authors:** Baukje Hemmes, Cécile R. L. P. N. Jeukens, Aliaa Al-Haidari, Paul A. M. Hofman, Ed S. vd Linden, Peter R. G. Brink, Martijn Poeze

**Affiliations:** Network Acute Care Limburg, Maastricht University Medical Center, Maastricht, The Netherlands; Department of Radiology and Nuclear Medicine, Maastricht University Medical Center, Maastricht, The Netherlands; Department of Surgery, Maastricht University Medical Center, Maastricht, The Netherlands; NUTRIM, School of Nutrition and Translational Research in Metabolism, Maastricht University, Maastricht, The Netherlands

**Keywords:** CT scan, Image quality, Spineboard, Vacuum mattress, Headblocks

## Abstract

Trauma patients at risk for, or suspected of, spinal injury are frequently transported to hospital using full spinal immobilisation. At the emergency department, immobilisation is often maintained until radiological work-up is completed. In this study, we examined how these devices for spinal stabilization influence visual image quality. Image quality was judged for both patient CT scans and phantom CT scans. CT scans of 217 patients were assessed retrospectively by two radiologists for visual scoring of image quality, scoring both quantity and impact of artifacts caused by the immobilization devices. For the phantom CT scans, eight set-ups were made, using a vacuum mattress without headblocks and a rigid and a soft-layered spineboard without headblocks, with standard soft-foam headblocks, or with new design headblocks. Overall, artifacts were found in 67 % of CT scans of patients on immobilization devices, which hampered diagnosis in 10 % of the cases. In the phantom CT scans, artifacts were present in all set-ups with one or more devices present and were seen in 20 % of all scan slices. The presence of headblocks resulted in more artifacts in both the patient CT scans and the phantom CT scans. Considerable effort should therefore be made to adjust the design of the immobilization devices and to remove the headblocks before CT scans are made.

## Introduction

In trauma patients at risk of spinal injury or brain injury, immobilisation using a spineboard and headblocks or vacuum mattress has become the gold standard in prehospital care, including transport of patients to the hospital [1–4]. Although physicians are advised to remove these devices as soon as possible [1–3], many patients undergo clinical and radiological evaluation in the emergency department and/or radiology department to rapidly assess life threatening injuries while still on the device [5, 6], and removal of the device is often postponed until after x-ray and CT imaging is done [4, 5].

In recent years, devices for spinal immobilisation have received renewed attention, focusing on patient (dis)comfort [7, 8] and functional restrictions [9–11], resulting in the redesign of some devices. Together with the traditional devices such as the rigid spineboard and the vacuum mattress, there is now a wide range of devices for spinal immobilisation available to choose from. However, all of these devices are made of plastics and foam and contain cut-outs or, in case of the vacuum mattress, folds, which can create artifacts on the CT scans [12–14], hampering identification or exclusion of pathology. A number of studies have described how misinterpreting artifacts as pathology can affect patient treatment [15–18].

To our knowledge, no study has yet been published comparing a vacuum mattress and spineboards plus headblocks with regard to their effect on image quality. We therefore aim to gain insight in the number of artifacts caused by the various devices using both patient and phantom scans. Furthermore, we assess how often these hamper diagnosis in clinical practice. We hypothesize that the soft foam headblocks and the rigid spineboard to cause more objective and subjective image quality loss compared to the newer design headblocks and spineboard and the vacuum mattress.

## Materials and methods

### Patient data

We performed a retrospective study of the data of 241 consecutive blunt trauma patients in 2011, who underwent standardized automatic exposure-controlled (AEC) multidetector CT of the brain during their initial resuscitation. The institutional ethics board approved the study with informed consent waived. Depending on the nature of the accident and other (potential) injuries, the patients were presented for CT scan lying on a rigid spineboard (Millennia Backboard; Ferno-Washington, Wilmington, OH), a soft-layered spineboard [7], a vacuum mattress (RedVac, Radstadt, Austria), or no device, with either standard soft-foam headblocks (universal head immobilizer; Ferno-Washington Inc, Wilmington, OH), new design headblocks speedblocks; Laerdal Medical, Stavanger, Norway), or no headblocks. Patients with incomplete data sampling (*n* = 24) were excluded from analysis. A furthermore five patients who appeared to be scanned with the technically impossible combination of no spineboard plus soft foam headblocks were excluded from analysis, resulting in a total of 212 patients with complete data.

All patient CT scans were obtained using a Philips Brilliance 64 (Philips, Best, the Netherlands) with tube potential 120 kV, effective current time 179 mAs, beam collimation 64 × 0.9 mm, and AEC enabled. The presence of artifacts was judged based on the 5 mm reconstructions of the scans. Artifacts were defined as any disturbance in visual image quality, such as lines or streak artifacts, due to objects lying between the radiation beam and the receiver. Artifacts which could be attributed to (medical) implants or devices other than those for spinal immobilisation were not scored in this study.

For 5-mm reconstruction scans that were judged to show artifacts, the original 1 mm scans were used to assess impact of the artifacts on clinical judgment. If presence or exclusion of pathology could not be conclusively judged on the 1 mm scans due to the presence of artifacts, the artifacts were scored as hampering judgment. All scans were judged by a radiologist and a random sample of 25 % and all the scans was assessed by a second radiologist blinded from the assessment of the first observer.

### Phantom data

CT scans were obtained of the various devices for spinal immobilisation in combination with a head phantom (density equal to water) CT. All phantom scans were obtained using a Philips Brilliance 64 (Philips, Best, The Netherlands) with tube potential 120 kV, effective tube current 179 mAs, beam collimation 64 × 0.9 mm, and AEC disabled. All slices were judged on the 1 mm scans on whether artifacts were present or not, thereby obtaining information not only on the presence but also on the extent of the artifacts. Both an experienced trauma surgeon and an experienced radiologist judged all scans.

### Statistical analyses

Statistical analysis was performed using the Statistical Package for Social Sciences (SPSS; IBM Chicago, IL), version 20.0.0. Non-parametric data are compared using Kruskal-Wallis testing for overall differences with significance assumed at *P* < 0.05 and Mann–Whitney *U* test for differences between devices with significance after Bonferroni correction assumed at (*P* < 0.05/number of tests). Parametric data are compared using one-way ANOVA for differences between devices with significance assumed at *P* < 0.05.

## Results

### Patient data

Of the 217 patients analyzed, 68 % were male, median age was 48 years (range 0–95; standard deviation 21.9). Most patients (65 %) were scanned lying on a rigid spineboard with new design headblocks. Eleven percent of patients were scanned lying on the soft-layered spineboard with or without headblocks, and 12 % of patients were scanned without any device in place (Table 1). A review of electronic patient files showed that 31 % of patients had abnormalities of face, skull, and/or brain diagnosed on CT.

**Table 1:** Artifacts in CT scans of the head of trauma patients (*n* = 212)

	Number of patients	Scans with artifacts	Scans with artifacts hampering clinical judgment
No spineboard, no headblocks	25	9 (36 %)	1 (4 %)
Rigid spineboard, no headblocks	12	5 (42 %)	1 (8 %)
Rigid spineboard, standard soft-foam headblocks	10	7 (70 %)	1 (10 %)
Rigid spineboard, new design headblocks	140	106 (76 %)	15 (11 %)
Soft-layered spineboard, no headblocks	5	2 (40 %)	0 (−)
Soft-layered spineboard, standard soft-foam headblocks	18	14 (78 %)	2 (11 %)
Soft-layered spineboard, new design headblocks	0	–	–
Vacuum mattress	2	1 (50 %)	1 (50 %)
Overall	212 (100 %)	144 (68 %)	21 (10 %)

**Fig. 1:**
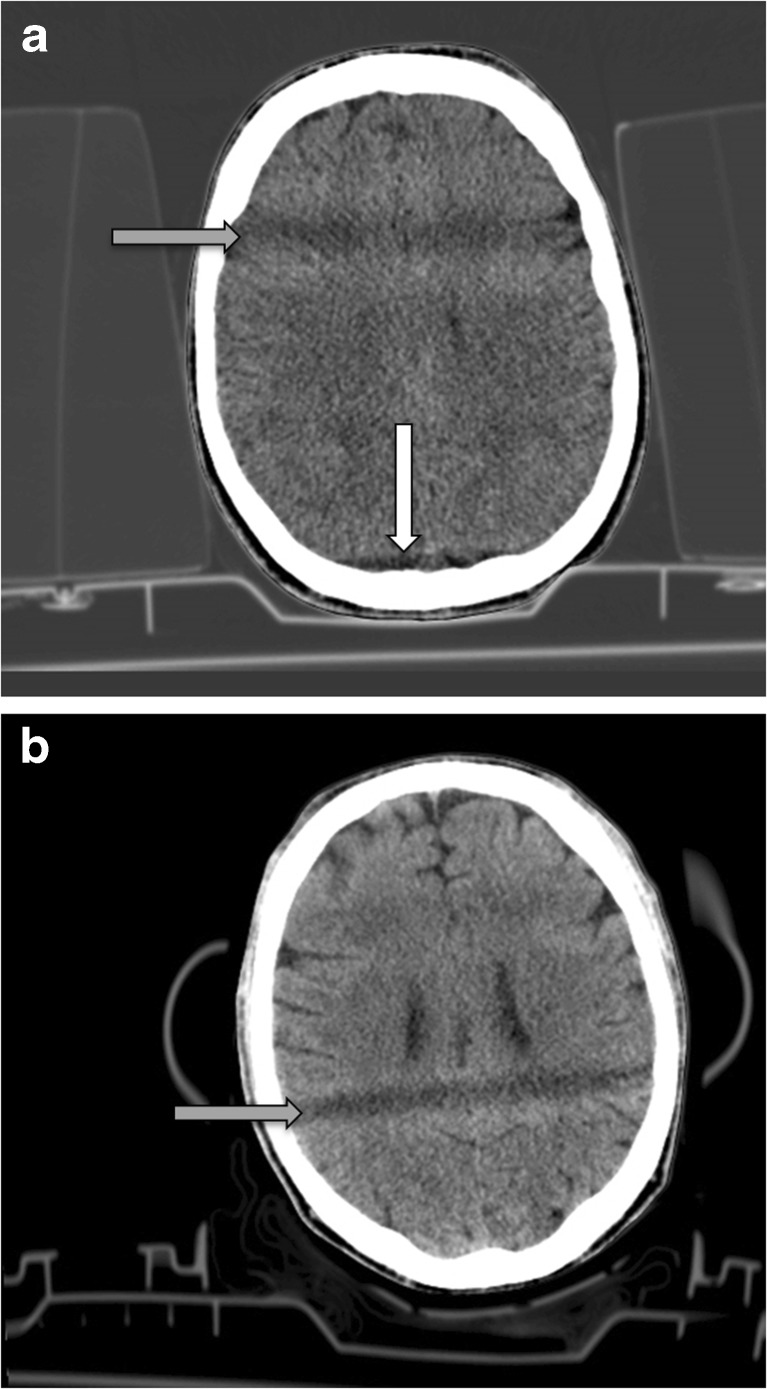
**a** Patient CT scan with artifact caused by headblocks. Set up indicates rigid spineboard plus soft foam headblocks. *Grey arrow* indicates hypodensity caused by material to air transition (earholes) in the headblocks. *White arrow* indicates line artifact cause by the base plate of the headblocks. **b** Patient CT scan with artifact caused by new design headblocks. Set-up indicates rigid spineboard plus new design headblocks. *Grey arrow* indicates shade caused by material to air transition in the headblocks

In the trauma patients, the percentage of scans with artifacts varied between 40 and 78 %, depending on the devices used (Table 1). Figure 1 presents two examples of typical artifacts encountered. Scans with only a spineboard or vacuum mattress and no headblocks showed artifacts in 40–50 % of the scans. Scans with a spineboard plus headblocks showed artifacts in 70–78 % of the scans. Interrater reliability was moderate (Cohen’s Kappa = 0.53, *p* < 0.01) for the number of scans with an artifact; differences were resolved through discussion. One-way independent ANOVA showed significant differences between the groups (*F*(7, 209) = 3.678, *p* < 0.01). Overall, the artifacts hampered clinical judgment in 10 % of the scans. In four patients, a conclusive diagnosis could not be made due to the presence of artifacts, although this did not change the treatment of the head trauma.

In 47 patients, a follow up CT scan was made in accordance with guidelines, either as a regular control scan after 6 to 24 h or at any time due to increased complaints. In five of these patients, subtle abnormalities in the primary scan which were at the time interpreted as possible injuries (none of clinical significance) were retrospectively judged as artifacts based on results of the repeat scan.

### Phantom data

**Fig. 2:**
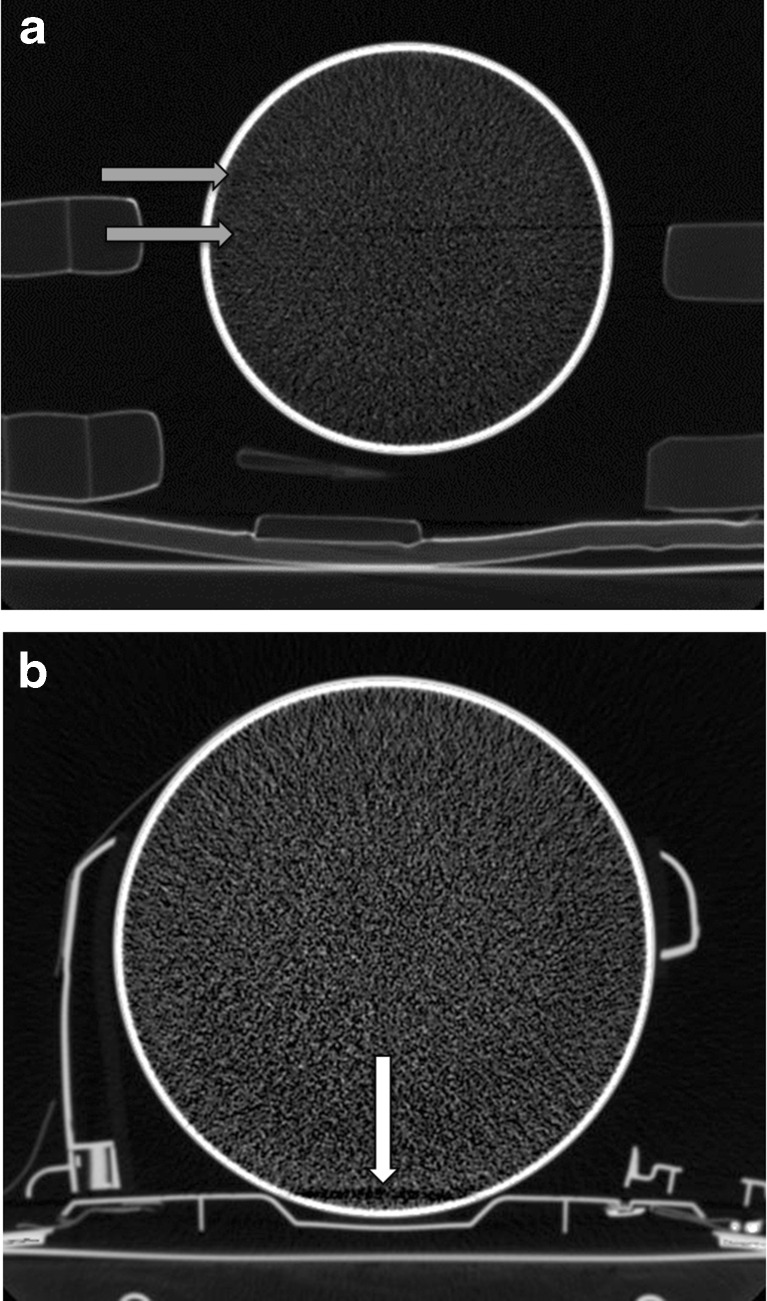
**a** Phantom CT scan with artifact caused by headblocks. Set-up indicates rigid spineboard plus soft-foam headblocks. *Grey arrows* indicate artifacts caused by matter to air transition of the headblocks. **b** Phantom CT scan with artifact caused by headblocks baseplate. Set-up indicates rigid spineboard plus new design headblocks. *White arrow* indicates artifact caused by base plate

In the phantom study, a mean of one in every five CT scan slices was disturbed by artifacts, with the percentages varying from 0 to 68 % depending on the immobilisation devices used (Table 2). Figure 2a, b shows examples of typical artifacts. Kruskal-Wallis test for non-parametric data showed a significant overall effect for differences between set-ups in artifact percentages (H(6) = 484.9, *p* < 0.001). Mann–Whitney tests with Bonferroni correction indicated that using headblocks significantly increased the number of slices with artifacts for both spineboards, with the standard soft-foam headblocks producing more artifacts compared to the new design headblocks. The vacuum mattress caused significantly less artifacts compared to either spineboard plus soft-foam headblocks. Interrater reliability was high for the presence of artifacts (Cronbach’s alpha = 0.88 with *r* = 0.78). The majority of differences in judgment were related to the extent of the artifacts.

**Table 2:** Artifacts in the phantom study; judgment of slices with artifacts

Condition	Number of slices	Average number and percentage of slices with artifacts	Alpha	*R*
Rigid spineboard, no headblocks	168	0 (0 %)	^a^	^b^
Rigid spineboard, standard soft-foam headblocks	151	103 (68 %)	0.75	0.61 (*p* < 0.01)
Rigid spineboard, new design headblocks	196	28 (14 %)	0.88	0.79 (*p* < 0.01)
Soft-layered spineboard, no headblocks	170	1 (1 %)	^a^	^b^
Soft-layered spineboard, standard soft-foam headblocks	172	105 (61 %)	0.80	0.66 (*p* < 0.01)
Soft-layered spineboard, new design headblocks	165	18 (11 %)	0.98	0.97 (*p* < 0.01)
Vacuum mattress, no headblocks	178	8 (4 %)	^a^	^b^
Overall	1200	262 (22 %)	0.88	0.78 (*p* < 0.01)

^a^Alpha cannot be calculated because the scale had zero variance for one or both of the judges

^b^Correlation cannot be calculated because at least one of the variables is constant

## Discussion

Our study results show that devices used for spinal immobilization negatively influence image quality in CT scans of the head. The presence of headblocks, especially the soft-foam headblocks, resulted in the highest degradation of image quality in both the patient scans and the phantom scans. In the patient scans, the presence of artifacts hampered clinical judgment in 10 % of cases with artifacts, although in none of the cases this resulted in a treatment change.

Earlier studies have investigated the influence of immobilisation devices on image quality. Miller et al. [13] showed that x-rays of the thorax in trauma patients can be difficult to interpret due to artifacts caused by the spineboard. Schou et al. [14] compared seven different types of vacuum mattress using an x-ray phantom, concluding that the artifacts caused by these devices may obscure fracture lines. Loewenhardt et al. [19] compared nine spineboard and vacuum matresses using a CTDI phantom, judging the devices according to severity of the artifacts. They concluded that the rigid spineboard identical to the one used in our study and the vacuum mattress produced no artifacts. Although we found similar results in our phantom study, the CT scans in actual patients did show artifacts attributable to the rigid spineboard. These findings indicate that in addition to evaluating image quality using a phantom model, clinical importance in patient CT scans is necessary to evaluate the impact on visual image quality impairment. Furthermore, artifacts caused by headblocks are an underrated issue and are mentioned only sporadically [20]. Our study is the first to systematically evaluate the impact of headblocks on the radiological image quality. We found the headblocks to add significantly to the number of artifacts seen on CT scans.

Both Du Plessis [17] and Mathur [18] presented cases showing how artifacts in CT scans of the brain can result in unnecessary additional diagnostics and medical treatment. In our series, in four patients, the diagnosis was adjusted based on results of the follow-up scan, although treatment remained unchanged.

In our study, all devices caused artifacts in the patient CT scans. Since these artifacts have shown to hamper clinical judgment in some cases, effort should be made to avoid the artifacts. This can be achieved by removing the devices before the CT scan is made or by improving the radiological properties of the devices. Implementation of changes in emergency care protocols remains difficult [2, 21, 22], indicating that propagating removal of the devices cannot be used as the sole means to reduce the occurrence of artifacts in CT scans. An added solution to limiting artifacts lies in improving the devices by maximizing radiopacity, minimizing sharp corners, and reducing material to air transitions at the location of the head.

Some remarks of this study are noted. Two important issues in medical radiation are image quality and radiation dose. Although in this study, we only looked at image quality, it is important to recognize the influence the devices have on the radiation dose patients are exposed to. The devices absorb part of the radiation, thereby increasing image dose, although there is also a certain shielding effect. These influences of the devices are discussed in detail by Hemmes et al. elsewhere [23]. In this study, we focussed on the image quality in relation to immobilisation devices. A direct comparison of the various immobilisation devices in the same patient would be advantageous but is not feasible in trauma care. Therefore, we studied a sufficient number of patients for interindividual comparison and executed a direct comparison of the devices using a phantom. In the phantom study, we only evaluated set-ups which were in line with current standards [24, 25]; however, in the patient study, we also encountered some scans with the use of headblocks but no spineboard. In these patients, the lumbar and thoracic spine had been cleared by clinical assessment; only the c-spine needed to be evaluated by CT scan. Furthermore, phantom CT scans were judged by a surgeon and a radiologist, while patient scans were judged by two radiologists. In our clinic, CT scans of trauma patients are always primarily judged (at the time of survey at the ED) by both the treating surgeon and a radiologist. We therefore deemed it appropriate to get an insight in how much artifacts both specialists see on the scans. Final judgment of the scans is in our clinic always made by a radiologist. We therefore asked two radiologists go make a judgment on artifacts on patient CT scans and also judge whether these artifacts hamper clinical judgment. The studies were non-blinded. Although the judges were not informed explicitly which immobilization devices were present, this could be deduced from the CT images. However, artifacts were also found in the CT scans that were obtained without devices present, and agreement between judges was high. Finally, all CT scans were obtained using the same Philips Brilliance 64 scanner. Although it could be argued that the type of scanner has an impact on visual image quality, for this study this impact will be limited. The type of artifact described in our study is caused by beam hardening due to energy absorption by the devices. This explains why using a spineboard combined with headblocks results in more artifacts compared to using a spineboard without headblocks. The difference in extent of artifacts between the soft foam and the new design headblocks depends on the size of the devices, with the larger 26-cm-long soft foam headblocks causing artifacts over more slices compared to the smaller 19 cm long new design headblocks. Due to these innate factors of artifact occurrence, using other CT scanners will likely show similar outcomes.

## Conclusion

Removing the headblocks before making a CT scan of the head significantly improves both objective and subjective image quality. Considerable effort should therefore be made to remove the headblocks and modulate newer versions of spinal immobilization devices.

## References

[1] Cooke MW (1998). Use of the spinal board within the accident and emergency department. J Accid Emerg Med.

[2] Lerner EB, Moscati R (2000). Duration of patient immobilization in the ED. Am J Emerg Med.

[3] Malik MHA, Lovell ME (2003). Current spinal board usage in emergency departments across the UK. Injury.

[4] Stagg MJ, Lovell ME (2008). A repeat audit of spinal board usage in the emergency department. Injury.

[5] El-Khoury GY, Kathol MH, Daniel WW (1995). Imaging of acute injuries of the cervical spine: value of plain radiography, CT, and MR Imaging. AJR.

[6] France JC, Bono CM, Vaccaro AR (2005). Initial radiographic evaluation of the spine after trauma: when, what, where, and how to image the acutely traumatized spine. J Orthop Trauma.

[7] Hemmes B, Poeze M, Brink PRG (2010). Reduced tissue-interface pressure and increased comfort on a newly developed soft-layered long spineboard. J Trauma.

[8] Oomens CWJ, Zenhorst W, Broek M (2013). A numerical study to analyse the risk for pressure ulcer development on a spine board. Clin Biomech.

[9] Ay D, Aktas C, Yesilyurt S, Sarikaya S, Cetin A, Ozdogan ES (2011). Effects of spinal immobilization devices on pulmonary function in healthy volunteer individuals. Turk J Trauma Emerg Surg.

[10] Ng I, Lim J, Wong HB (2004). Effects of head posture on cerebral hemodynamics; its influences on intracranial pressure, cerebral perfusion pressure and cerebral oxygenation. Neurosurgery.

[11] White CCDR, Millin MG, Standards and Clinical Practice Committee, National Association of EMS Physicians (2014). EMS spinal precautions and the use of the long backboard - resource document to the position statement of the national association of EMS physicians and the American College of Surgeons committee on trauma. Prehosp Emerg Care.

[12] Daffner RH, Khoury MB (1987). Pseudofractures due to Nec-Loc cervical immobilization collar. Skelet Radiol.

[13] Miller JA, Mele C, Abu-Judeh H (1999). Significance of backboard artifacts on portable trauma series chest radiographs. Emerg Radiol.

[14] Schou J, Kiermayer H, Ummenhofer W, Herion H-P (2001). In search of the most suitable technique for truncal spinal immobilization with associated radiography. Eur J Emerg Med.

[15] Hayes M, Andronikou S, Prabhu S, Schulze O (2006). Multiplanar angles reconstructions of reconstructions - a dangerous practice using multidetector CT. Pediatr Radiol.

[16] Wells IT, Manghat N (2009). Re: A CT reconstruction artefact that mimics acute subdural haemorrhage. Clin Radiol.

[17] du Plessis A-M, Theron S, Andronikou S (2009). The effects of misinterpretation of an artefact on multidetector row CT scans in children. Pediatr Radiol.

[18] Mathur S, Gadde S, Koteyar SS (2008). Artefact misinterpretation on CT images of the head. Clin Radiol.

[19] Loewenhardt B, Huttinger R, Reinert M, Hering B, Rathjen T, Gries A (2014). Dose effects and image quality: Is there any influence by bearing devices in whole-body computed tomography in trauma patients?. Injury.

[20] Johnson RJ, Bury RW (2011). Cervical spine radiology. Anaesth Intensive Care Med.

[21] Hauswald M (2013). A re-conceptualisation of acute spinal care. Emerg Med J.

[22] Hauswald M, Braude D (2007). Diffusion of medical progress: early spinal immobilization in the Emergency Department. Acad Emerg Med.

[23] Hemmes B, Jeukens CRLPN, Kemerink GJ, Brink PRG, Poeze M (2016) Effect of spinal immobilisation devices on radiation exposure in conventional radiography and computed tomography. Emerg Rad 23:147–53 10.1007/s10140-015-1371-0 PMC4805719 26754428

[24] American College of Surgeons editor. Advanced Trauma Life Support. Student course manual, 9th ed. Chicago, IL 2012

[25] National Association of Emergency Medical Technicians editor. PHTLS: Prehospital Trauma Life Support. 8th ed. Jones & Bartlett Learning, 2014

